# Risk Categories in COVID-19 Based on Degrees of Inflammation: Data on More Than 17,000 Patients from the Spanish SEMI-COVID-19 Registry

**DOI:** 10.3390/jcm10102214

**Published:** 2021-05-20

**Authors:** Manuel Rubio-Rivas, Xavier Corbella, Francesc Formiga, Estela Menéndez Fernández, María Dolores Martín Escalante, Isolina Baños Fernández, Francisco Arnalich Fernández, Esther Del Corral-Beamonte, Antonio Lalueza, Alejandro Parra Virto, Emilia Roy Vallejo, José Loureiro-Amigo, Ana María Álvarez Suárez, Jesica Abadía-Otero, María Navarro De La Chica, Raquel Estévez González, Almudena Hernández Milián, María Areses Manrique, Julio César Blázquez Encinar, Amara González Noya, Ruth González Ferrer, María Pérez Aguilera, Ricardo Gil Sánchez, Jesús Millán Núñez-Cortés, José Manuel Casas-Rojo, on behalf of the SEMI-COVID-19 Network

**Affiliations:** 1 Department of Internal Medicine, Bellvitge University Hospital, Bellvitge Biomedical Research Institute-IDIBELL, University of Barcelona, 08907 Barcelona, Spain; xcorbella@bellvitgehospital.cat (X.C.); fformiga@bellvitgehospital.cat (F.F.); 2 Hestia Chair in Integrated Health and Social Care, School of Medicine, Universitat Internacional de Catalunya, 08017 Barcelona, Spain; 3 Department of Internal Medicine, San Pedro Hospital, 26006 Logroño, Spain; estelamf92@gmail.com; 4 Department of Internal Medicine, Hospital Costa del Sol, 29603 Marbella, Spain; mmartinescalante@gmail.com; 5 Department of Internal Medicine, Hospital Puerta de Hierro Hospital, 28220 Majadahonda, Spain; isolina6@hotmail.com; 6 Head of the Department of Internal Medicine, La Paz Hospital, 28046 Madrid, Spain; farnalich@salud.madrid.org; 7 Department of Internal Medicine, Royo Villanova Hospital, 50007 Zaragoza, Spain; esdcorral@gmail.com; 8 Department of Internal Medicine, 12 de Octubre University Hospital, 28041 Madrid, Spain; antonio.lalueza@salud.madrid.org; 9 Department of Internal Medicine, Gregorio Marañón Hospital, 28009 Madrid, Spain; alejandro.parra@salud.madrid.org (A.P.V.); jesus.millan@salud.madrid.org (J.M.N.-C.); 10 Department of Internal Medicine, La Princesa University Hospital, 28006 Madrid, Spain; eroyvallejo@gmail.com; 11 Department of Internal Medicine, Moisès Broggi Hospital, 08970 Sant Joan Despí, Spain; jose.loureiro.amigo@gmail.com; 12 Department of Internal Medicine, Cabueñes Hospital, 33203 Gijón, Spain; anaas_93@hotmail.com; 13 Department of Internal Medicine, Río Hortega University Hospital, 47014 Valladolid, Spain; jabadiao@saludcastillayleon.es; 14 Department of Internal Medicine, Nuestra Señora del Prado Hospital, 45600 Talavera de la Reina, Spain; maria.navachica@yahoo.com; 15 Department of Internal Medicine, Virgen de la Salud Hospital-Toledo University Hospital, 45004 Toledo, Spain; Raqueleg@gmail.com; 16 Department of Internal Medicine, Son Llatzer Hospital, 07005 Mallorca, Spain; adelapen@hsll.es; 17 Department of Internal Medicine, Santa Marina Hospital, 48004 Bilbao, Spain; MARIA.ARESESMANRIQUE@osakidetza.eus; 18 Department of Internal Medicine, Torrevieja University Hospital, 03186 Torrevieja, Spain; jcbencinar@gmail.com; 19 Department of Internal Medicine, Ourense University Hospital, 32005 Ourense, Spain; amara.gonzalez.noya@sergas.es; 20 Department of Internal Medicine, Tajo University Hospital, 41007 Toledo, Spain; ruthgferrer@gmail.com; 21 Department of Internal Medicine, Juan Ramón Jiménez University Hospital, 21005 Huelva, Spain; maghalit@gmail.com; 22 Department of Internal Medicine, La Fe Hospital, 46009 Valencia, Spain; rigilsan@gmail.com; 23 Department of Internal Medicine, Infanta Cristina University Hospital, 28981 Parla, Spain; jm.casas@gmail.com

**Keywords:** COVID-19, cytokine storm, prognosis, risk factors, mortality

## Abstract

(1) Background: The inflammation or cytokine storm that accompanies COVID-19 marks the prognosis. This study aimed to identify three risk categories based on inflammatory parameters on admission. (2) Methods: Retrospective cohort study of patients diagnosed with COVID-19, collected and followed-up from 1 March to 31 July 2020, from the nationwide Spanish SEMI-COVID-19 Registry. The three categories of low, intermediate, and high risk were determined by taking into consideration the terciles of the total lymphocyte count and the values of C-reactive protein, lactate dehydrogenase, ferritin, and D-dimer taken at the time of admission. (3) Results: A total of 17,122 patients were included in the study. The high-risk group was older (57.9 vs. 64.2 vs. 70.4 years; *p* < 0.001) and predominantly male (37.5% vs. 46.9% vs. 60.1%; *p* < 0.001). They had a higher degree of dependence in daily tasks prior to admission (moderate-severe dependency in 10.8% vs. 14.1% vs. 17%; *p* < 0.001), arterial hypertension (36.9% vs. 45.2% vs. 52.8%; *p* < 0.001), dyslipidemia (28.4% vs. 37% vs. 40.6%; *p* < 0.001), diabetes mellitus (11.9% vs. 17.1% vs. 20.5%; *p* < 0.001), ischemic heart disease (3.7% vs. 6.5% vs. 8.4%; *p* < 0.001), heart failure (3.4% vs. 5.2% vs. 7.6%; *p* < 0.001), liver disease (1.1% vs. 3% vs. 3.9%; *p* = 0.002), chronic renal failure (2.3% vs. 3.6% vs. 6.7%; *p* < 0.001), cancer (6.5% vs. 7.2% vs. 11.1%; *p* < 0.001), and chronic obstructive pulmonary disease (5.7% vs. 5.4% vs. 7.1%; *p* < 0.001). They presented more frequently with fever, dyspnea, and vomiting. These patients more frequently required high flow nasal cannula (3.1% vs. 4.4% vs. 9.7%; *p* < 0.001), non-invasive mechanical ventilation (0.9% vs. 3% vs. 6.3%; *p* < 0.001), invasive mechanical ventilation (0.6% vs. 2.7% vs. 8.7%; *p* < 0.001), and ICU admission (0.9% vs. 3.6% vs. 10.6%; *p* < 0.001), and had a higher percentage of in-hospital mortality (2.3% vs. 6.2% vs. 23.9%; *p* < 0.001). The three risk categories proved to be an independent risk factor in multivariate analyses. (4) Conclusion: The present study identifies three risk categories for the requirement of high flow nasal cannula, mechanical ventilation, ICU admission, and in-hospital mortality based on lymphopenia and inflammatory parameters.

## 1. Introduction

As of 6 February 2021, more than 105 million people have contracted SARS-CoV-2, and more than 2.3 million people have died from COVID-19. After 1 year of advances in the understanding of the disease, several risk factors have been recognized [1,2]. These include older age, male gender, certain comorbidities, and phenotypic clusters based on patient symptomatology [3,4,5,6,7,8,9]. Some analytical parameters have also been identified as poor prognostic factors and are related to the inflammatory state that patients present during the disease, the so-called cytokine storm, the most characteristic features of which are a decrease in the lymphocyte count and an increase in inflammatory parameters such as C-reactive protein (CRP), lactate dehydrogenase (LDH), ferritin, and D-dimer [10]. This accompanying inflammatory process marks the prognosis of COVID-19.

Our study aimed to identify three risk categories based on inflammatory parameters on admission.

## 2. Materials and Methods

### 2.1. Study Design, Patient Selection, and Data Collection

This is a retrospective cohort study with data on patients collected and followed-up from 1 March to 31 July 2020, from the nationwide Spanish SEMI-COVID-19 Registry. The characteristics of the patients included in this registry have been extensively described [11]. This is a multicenter, nationwide registry with over 150 hospitals registered so far. From 1 March to 31 July 2020, a total of 17,122 hospitalized patients diagnosed with COVID-19 on admission were included in the Registry. All included patients were diagnosed by polymerase chain reaction (PCR) test taken from a nasopharyngeal sample, sputum, or bronchoalveolar lavage. All patients’ symptoms were collected on admission. Likewise, blood values were collected on the first day of hospital admission and prior to receiving any type of therapy. The collection of data from each patient in terms of laboratory data, treatments, and outcomes was verified by the principal investigator of each center through the review of clinical records. The patients included in the present study had not received corticosteroid or anti-inflammatory therapy prior to hospital admission. To assess functional status prior to admission, the Barthel index was used: independent or mild dependence, 100–91; moderate dependence, 90–61; and severe dependence, ≤60.

All participating centers in the register received confirmation from the relevant Ethics Committees, including Bellvitge University Hospital (PR 128/20).

### 2.2. Categories of Risk

The three categories of low, intermediate, and high risk were determined by taking into consideration the terciles of the total lymphocyte count, and the CRP, LDH, ferritin, and D-dimer values taken at the time of admission. The low-risk category was defined when all parameters were in the first tercile. The high-risk category was defined when any of the parameters were in the third tercile. The intermediate risk category was defined when the defining conditions of low or high risk were not met.

### 2.3. Outcomes Definition

The primary outcome of the study was in-hospital mortality. The secondary outcomes were the requirement of high flow nasal cannula (HFNC), non-invasive mechanical ventilation (NIMV), invasive mechanical ventilation (IMV), or intensive care unit (ICU) admission.

### 2.4. Statistical Analysis

Categorical variables were expressed as absolute numbers and percentages. Continuous variables are expressed as mean plus standard deviation (SD) in the case of parametric distribution, or median (IQR) in the case of non-parametric distribution. Differences among groups were assessed using the chi-square test for categorical variables and ANOVA or the Kruskal–Wallis test, as appropriate, for continuous variables; *p*-values <0.05 indicated statistical significance.

For the assessment of risk factors for each of the outcomes, binary logistic regression was performed, including the three risk categories in the model. In multivariate analyses, variables with a significance of <0.10 in the univariate analyses plus age and sex were included. Subsequently, the differences in mortality were shown graphically using Kaplan–Meier curves, with their log-rank test (event: death; censored data: hospital discharge). Missing data were treated with multiple imputation.

Statistical analysis was performed using IBM SPSS Statistics for Windows, Version 26.0. Armonk, NY, USA, IBM Corp.

## 3. Results

### 3.1. General Data and Symptoms

A total of 17,122 patients were included in the study. Table 1 shows the three categories of low, intermediate, and high risk and their cut-off points. Table 2 shows the overall data for the three risk categories. Of note, members of the high-risk group were older (57.9 years vs. 64.2 vs. 70.4; *p* < 0.001) and more predominantly male (37.5% vs. 46.9% vs. 60.1%; *p* < 0.001). They had a higher degree of dependency prior to admission and certain comorbidities were more prevalent (arterial hypertension, dyslipidemia, diabetes mellitus, ischemic heart disease, chronic heart failure, chronic liver disease, severe chronic renal failure, cancer, and chronic obstructive pulmonary disease). In contrast, asthma was less prevalent in this group of patients.

Symptoms at the time of hospital admission are shown in Table 3. The high-risk group presented more frequently with fever (71.3% vs. 83% vs. 84.3%; *p* < 0.001), dyspnea (52.3% vs. 51.1% vs. 59.1%; *p* < 0.001), and vomiting (3.4% vs. 7.8% vs. 8%; *p* = 0.007).

### 3.2. Lab Tests among Categories

The lab test results (on admission) for the three categories are shown in Table 4.

### 3.3. Outcomes

The outcomes by risk category are shown in Table 5. All outcomes were more prevalent in the high-risk group. Thus, these patients more frequently required HFNC (3.1% vs. 4.4% vs. 9.7%; *p* < 0.001), NIMV (0.9% vs. 3% vs. 6.3%; *p* < 0.001), IMV (0.6% vs. 2.7% vs. 8.7%; *p* < 0.001), or ICU admission (0.9% vs. 3.6% vs. 10.6%; *p* < 0.001), and had a higher percentage of in-hospital mortality (2.3% vs. 6.2% vs. 23.9%; *p* < 0.001).

**Figure 1:**
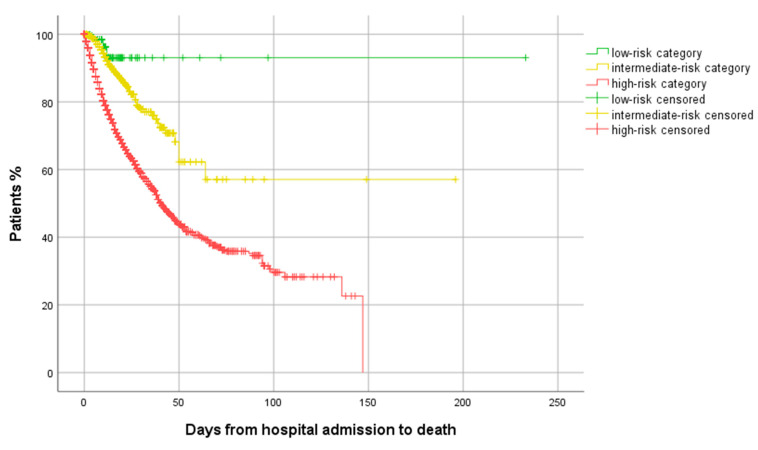
Time to death for the three categories (Log rank 325.6 *p* < 0.001).

**Figure 2:**
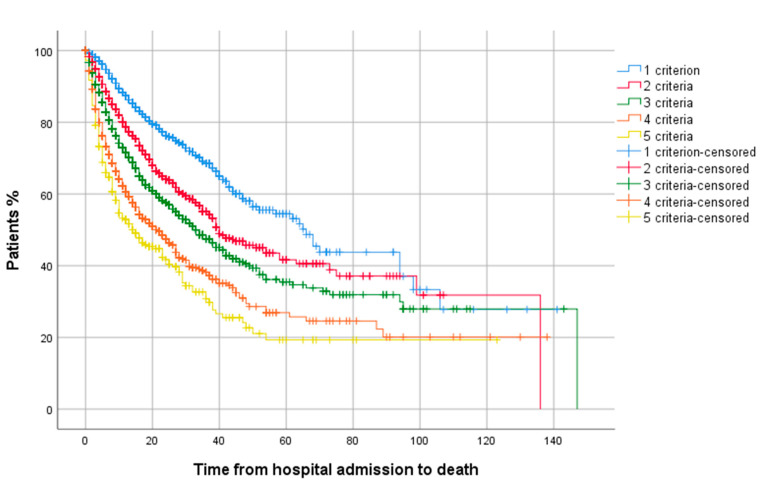
Time to death for high-risk patients according to the number of high-risk criteria.

Figure 1 shows the survival curves for the three risk categories. Figure 2 shows the survival curves in high-risk patients depending on the number of high-risk criteria (lymphocytes, CRP, LDH, ferritin, or D-dimer) above the predetermined threshold. Table S1 shows the outcomes in high-risk patients depending on how many high-risk parameters each patient had.

### 3.4. Risk Factors for HFNC, NIMV, IMV, ICU Admission and in-Hospital Mortality

The multivariate binary logistic regression shown in Table 6, and in supplemental files, Tables S2–S5, identify the three risk categories as an independent risk factor for HFNC, NIMV, MIV, ICU admission, and in-hospital death. Taking the low-risk category as a reference, the attributed risk of in-hospital death was OR = 1.91 (0.9–4.05; *p* = 0.093) for the intermediate risk category and 7.31 (3.49–15.28; *p* < 0.001) for the high-risk category.

## 4. Discussion

The present study identifies three risk categories based on degrees of lymphopenia and inflammatory parameters present on hospital admission as part of the host-immune response and cytokine storm that accompanies severe COVID-19 [10]. Since the beginning of the pandemic, inflammation has been synonymous with clinical complications during admission and has ultimately increased the likelihood of death. This study not only confirms this observation, but also defines three clearly differentiated prognostic groups, both in terms of resource use (HFNC, NIMV, IMV) and in terms of ICU admission or in-hospital death. Furthermore, our study clearly and concisely describes the cut-off points that define each of these categories so that the research can inform clinical practice.

The high-risk category presents socio-demographic and comorbidity characteristics already recognized as poor prognostic factors [3,4,5,6,7,8,9]. There is no definitive answer to date as to why older patients, especially men and those with certain comorbidities, become more inflamed, but what is clear is that such patients’ inflammatory response is certainly greater. Nor is it surprising that these older men present more frequently with fever, dyspnea, and vomiting. Such symptoms may indicate phenotypic clusters C1 and C4, previously described by our group as poor prognostic factors [9].

Based on the figures for outcomes by category, the need to maintain hospital admission for patients in the low-risk category should be carefully considered. We believe that close outpatient follow-up would be more appropriate for such patients. Although results in all outcomes are worse for the intermediate-risk category with respect to the low-risk category, the most substantial changes occur in the high-risk category. We believe it is crucial to detect patients belonging to this category at the outset and take more intensive therapeutic management measures.

It is evident from the results obtained that it is not only important whether patients belong to the high-risk category or not, but also how many high-risk parameters are fulfilled. In this regard, it is still unclear whether one of the analyzed parameters is of greater importance than the others.

To date, guidelines, protocols, and trials are overly focused on oxygenation/ventilation status. Respiratory status is at the end of the slope of different causes, not only COVID-19 (obesity, previous pulmonary pathologies, hospital resources, etc). The cornerstone of any COVID-19 protocol should be inflammation. It precedes respiratory deterioration and has a remarkably high predictive power, given the results of the present study. Another factor to be considered in trials is assessing the effectiveness of anti-inflammatory/immunosuppressive drugs.

We believe that the usefulness of this risk stratification strategy is that it is not only prognostic in allowing us to identify those patients at greatest risk; this study can also serve as a generator of hypothesis to be confirmed in prospective studies on drug regimens [12,13]. Defining the target of patients likely to improve with such treatments has been the fundamental question for some time now, and we believe that these risk categories can help to define it. In fact, the next step in our research is to precisely explore this question. We believe that identification of these patients is a necessary step that will shed light on many uncertainties to date and will explain the discrepancies in the tocilizumab trials in COVID-19 in recent months [14,15,16,17].

In order to understand the risk factors for HFNC, NIMV, or IVM use, or ICU admission, it should be taken into account that some paradoxical “protective” factors in the logistic regression (degree of dependency in daily tasks, presence of severe comorbidities, etc.) are not such. This is because there is a limitation on the critical care therapeutic efforts in very old and highly dependent patients with severe comorbidities, in whom the more likely predicted outcome will result in mortality.

The present study has several strengths. Most notably, the large sample size from a consolidated national registry. In addition, the inclusion of first, second, and third level hospitals provides us with a broader picture of the pandemic in our territory.

As regards limitations, there is a notable predominance of Caucasian population in our registry and less representation of other ethnic groups, as reported previously [11]. In addition, we would have liked to include data referring to interleukin 6 as part of the panel of inflammatory parameters assessed, but, unfortunately, the high percentage of missing values in the registry did not allow us to do so. Finally, the registry allows us to identify patients with active cancer, but not if they were undergoing chemotherapy or active immunotherapy. Likewise, for the present study, we do not have data on body mass index or immunosuppressive treatments administered prior to admission.

## 5. Conclusions

The present study identifies three risk categories for HFNC, NIMV, or IMV use, ICU admission, and in-hospital mortality based on degrees of lymphopenia and inflammatory parameters that show severe COVID-19 patients on hospital admission. We believe their early identification and appropriate clinical management based on this risk stratification strategy may be crucial in improving the prognosis of COVID-19 patients requiring hospitalization.

## Figures and Tables

**Table 1:** Categories of risk.

	Low Risk	Intermediate Risk	High Risk
Lymphocyte count ×10^6^/L	>1150	760–1150	<760
CRP (mg/L)	<31.8	31.8–101.5	>101.5
LDH (U/L)	<271	271–394	>394
Ferritin (mcg/L)	<572.9	572.9–1359.9	>1359.9
D-dimer (ng/mL)	<539	539–1580	>1580

CRP: C-reactive protein. LDH: lactate dehydrogenase. The colors in the table indicate the risk traffic light in 3 categories: low risk (green), intermediate risk (yellow), and high risk (red).

**Table 2:** General data for the three categories.

	Low Risk	Intermediate Risk	High Risk	*p*-Value
N	352	3018	13,752	
Age, median (IQR)	57.9 (43.7–71.3)	64.2 (51.7–76.5)	70.4 (57.8–80.3)	<0.001
Gender (males)	132 (37.5)	1416 (46.9)	8264 (60.1)	<0.001
Days from onset to admission, median (IQR)	6 (3–10)	7 (4–9)	6 (3–9)	0.005
Smoking behaviour				<0.001
Never smoker	254 (72.2)	2201 (72.9)	9485 (69)	
Former smoker	71 (20.2)	664 (22)	3582 (26)	
Current smoker	27 (7.7)	153 (5.1)	685 (5)	
Degree of dependency				<0.001
None or mild	314 (89.2)	2595 (86)	11,423 (83.1)	
Moderate	24 (6.8)	231 (7.7)	1329 (9.7)	
Severe	14 (4)	192 (6.4)	1000 (7.3)	
Arterial hypertension	130 (36.9)	1365 (45.2)	7260 (52.8)	<0.001
Dyslipidemia	100 (28.4)	1118 (37)	5584 (40.6)	<0.001
Diabetes mellitus	42 (11.9)	516 (17.1)	2822 (20.5)	<0.001
Ischaemic cardiopathy	13 (3.7)	196 (6.5)	1151 (8.4)	<0.001
Chronic heart failure	12 (3.4)	158 (5.2)	1045 (7.6)	<0.001
Chronic liver disease	4 (1.1)	91 (3)	537 (3.9)	0.002
Severe chronic renal failure	8 (2.3)	108 (3.6)	920 (6.7)	<0.001
Cancer	23 (6.5)	216 (7.2)	1521 (11.1)	<0.001
COPD	20 (5.7)	163 (5.4)	972 (7.1)	0.003
Asthma	40 (11.4)	279 (9.2)	895 (7.1)	<0.001
OSAS	16 (4.5)	188 (6.2)	839 (6.1)	0.455

IQR: interquartile range. COPD: chronic obstructive pulmonary disease. OSAS: obstructive sleep apnea syndrome. Severe chronic renal failure: Creatinin >300 mg/dL or dyalisis.

**Table 3:** Symptoms and physical examination upon admission for the three categories.

	Low Risk	Intermediate Risk	High Risk	*p*-Value
Cough, n (%)	258 (73.3)	2269 (75.2)	9950 (72.4)	0.007
Arthromyalgias, n (%)	108 (30.7)	1027 (34)	4011 (29.2)	<0.001
Ageusia, n (%)	39 (11.1)	343 (11.4)	1070 (7.8)	<0.001
Anosmia, n (%)	43 (12.2)	303 (10)	926 (6.7)	<0.001
Sore throat, n (%)	50 (14.2)	344 (11.4)	1237 (9)	<0.001
Headache, n (%)	58 (16.5)	448 (14.8)	1499 (10.9)	<0.001
Fever, n (%)	251 (71.3)	2506 (83)	11,592 (84.3)	<0.001
Dyspnea, n (%)	184 (52.3)	1542 (51.1)	8121 (59.1)	<0.001
Diarrhea, n (%)	83 (23.6)	790 (26.2)	3224 (23.4)	0.006
Vomiting, n (%)	12 (3.4)	235 (7.8)	1097 (8)	0.007
Abdominal pain, n (%)	26 (7.4)	195 (6.5)	894 (6.5)	0.795
Heart rate, bpm median (IQR)	85.5 (75–98)	85 (75–97)	88 (77–100)	<0.001
Respiratory rate >20 rpm, n (%)	45 (12.8)	579 (19.2)	4809 (35)	<0.001

IQR: interquartile range.

**Table 4:** Lab tests upon admission for the three categories.

	Low Risk	Intermediate Risk	High Risk	*p*-Value
Lymphocytes ×10^6^/L, median (IQR)	1605 (1340–2100)	1180 (960–1500)	860 (600–1200)	<0.001
CRP mg/L, median (IQR)	6.3 (2.2–14.9)	30 (10.7–57)	81.5 (28.2–151)	<0.001
LDH U/L, median (IQR)	203 (175–230)	261 (213–312)	360 (267–486.4)	<0.001
Ferritin mcg/L, median (IQR)	220.3 (103.5–371)	534 (259.2–891)	1144.7 (532–1834.5)	<0.001
D-dimer ng/mL, median (IQR)	286.5 (210.8–400)	490 (280–776.3)	1131 (510–4347.8)	<0.001

CRP: C-reactive protein. LDH: lactate dehydrogenase. IQR: interquartile range.

**Table 5:** Outcomes for the three categories.

	Low Risk	Intermediate Risk	High Risk	*p*-Value
HFNC, n (%)	11 (3.1)	132 (4.4)	1319 (9.7)	<0.001
NIMV, n (%)	3 (0.9)	90 (3)	857 (6.3)	<0.001
IMV, n (%)	2 (0.6)	80 (2.7)	1195 (8.7)	<0.001
ICU admission, n (%)	3 (0.9)	109 (3.6)	1453 (10.6)	<0.001
In-hospital mortality, n (%)	8 (2.3)	186 (6.2)	3291 (23.9)	<0.001

HFNC: high Flow nasal cannula. NIMV: non-invasive mechanical ventilation. IMV: invasive mechanical ventilation. ICU: intensive care unit.

**Table 6:** Risk factors of in-hospital mortality.

	Univariate Analysis		Multivariate Analysis	
	OR (95% CI)	*p*-Value	OR (95% CI)	*p*-Value
Age	1.08 (1.08–1.09)	<0.001	1.07 (1.07–1.08)	<0.001
Gender (female)	0.76 (0.70–0.82)	<0.001	0.67 (0.61–0.74)	<0.001
Smoking behaviour				
Never smoker	1 ref.		1 ref.	
Former smoker	1.57 (1.45–1.70)	<0.001	1.12 (1.00–1.24)	0.052
Current smoker	1.06 (0.89–1.26)	0.540	1.27 (1.03–1.58)	0.025
Degree of dependency				
None or mild	1 ref.		1 ref.	
Moderate	4.20 (3.77–4.69)	<0.001	1.60 (1.41–1.83)	<0.001
Severe	4.99 (4.42–5.63)	<0.001	2.01 (1.73–2.33)	<0.001
Arterial hypertension	2.77 (2.56–3.00)	<0.001	-	NS
Dyslipidemia	1.77 (1.64–1.91)	<0.001	-	NS
Diabetes mellitus	1.97 (1.81–2.15)	<0.001	1.21 (1.09–1.34)	<0.001
Ischaemic cardiopathy	2.41 (2.14–2.71)	<0.001	1.25 (1.08–1.44)	0.002
Chronic heart failure	3.32 (2.94–3.74)	<0.001	-	NS
Chronic liver disease	1.49 (1.24–1.78)	<0.001	1.32 (1.07–1.63)	0.011
Severe chronic renal failure	3.19 (2.80–3.62)	<0.001	1.50 (1.29–1.76)	<0.001
Cancer	2.04 (1.83–2.27)	<0.001	1.54 (1.35–1.75)	<0.001
COPD	2.53 (2.24–2.87)	<0.001	-	NS
Asthma	0.62 (0.52–0.73)	<0.001	-	NS
OSAS	1.36 (1.18–1.57)	<0.001	-	NS
Respiratory rate > 20 rpm	3.91 (3.62–4.22)	<0.001	3.21 (2.94–3.51)	<0.001
Three risk categories				
Low risk	1 ref.		1 ref.	
Intermediate risk	2.82 (1.38–5.78)	0.005	1.91 (0.90–4.05)	0.093
High risk	13.53 (6.7–27.30)	<0.001	7.31 (3.49–15.28)	<0.001

NS: Not significant. COPD: chronic obstructive pulmonary disease. OSAS: obstructive sleep apnea syndrome.

## Data Availability

Restrictions apply to the availability of these data. Data was obtained from SEMI-COVID-19 registry and are available from the authors with the permission of SEMI-COVID-19 registry.

## Supplementary Materials

The following are available online at https://www.mdpi.com/article/10.3390/jcm10102214/s1, Table S1. Outcomes in high-risk patients according to the number of high-risk criteria; Table S2. Risk factors of the requirement of HFNC; Table S3. Risk factors of the requirement of NIMV; Table S4. Risk factors of the requirement of IMV; Table S5. Risk factors of ICU admission; Table S6. Matrix of correlations between prognostic factors.

## References

[1] Zhou F., Yu T., Du R., Fan G., Liu Y., Liu Z., Xiang J., Wang Y., Song B., Gu X. (2020). Clinical course and risk factors for mortality of adult inpatients with COVID-19 in Wuhan, China: A retrospective cohort study. Lancet.

[2] Novel Coronavirus Pneumonia Emergency Response Epidemiology Team (2020). The Epidemiological Characteristics of an Outbreak of 2019 Novel Coronavirus Diseases (COVID-19)—China, 2020. China CDC Weekly.

[3] Grasselli G., Zangrillo A., Zanella A., Antonelli M., Cabrini L., Castelli A., Cereda D., Coluccello A., Foti G., Fumagalli R. (2020). Baseline Characteristics and Outcomes of 1591 Patients Infected With SARS-CoV-2 Admitted to ICUs of the Lombardy Region, Italy. JAMA.

[4] Du R.-H., Liang L.-R., Yang C.-Q., Wang W., Cao T.-Z., Li M., Guo G.-Y., Du J., Zheng C.-L., Zhu Q. (2020). Predictors of Mortality for Patients with COVID-19 Pneumonia Caused by SARS-CoV-2: A Prospective Cohort Study. Eur. Respir. J..

[5] Rodilla E., López-Carmona M.D., Cortes X., Cobos-Palacios L., Canales S., Sáez M.C., Escudero S.C., Rubio-Rivas M., Manglano J.D., Castro S.J.F. (2020). Impact of arterial stiffness on all-cause mortality in patients hospitalized with COVID-19 in Spain. Hypertension.

[6] Pérez-Belmonte L.M., Torres-Peña J.D., López-Carmona M.D., Ayala-Gutiérrez M.M., Fuentes-Jiménez F., Huerta L.J., Muñoz J.A., Rubio-Rivas M., Madrazo M., Garcia M.G. (2020). Mortality and other adverse outcomes in patients with type 2 diabetes mellitus admitted for COVID-19 in association with glucose-lowering drugs: A nationwide cohort study. BMC Med..

[7] Ramos-Rincon J.-M., Buonaiuto V., Ricci M., Martín-Carmona J., Paredes-Ruíz D., Calderón-Moreno M., Rubio-Rivas M., Beato-Pérez J.-L., Arnalich-Fernández F., Monge-Monge D. (2021). Clinical Characteristics and Risk Factors for Mortality in Very Old Patients Hospitalized with COVID-19 in Spain. J. Gerontol. Ser. A Boil. Sci. Med. Sci..

[8] Wang B., Li R., Lu Z., Huang Y. (2020). Does comorbidity increase the risk of patients with covid-19: Evidence from meta-analysis. Aging.

[9] Rubio-Rivas M., Corbella X., Mora-Luján J.M., Loureiro-Amigo J., Sampalo A.L., Bergua C.Y., Atiénzar P.J.E., García L.F.D., Ferrer R.G., Canteli S.P. (2020). Predicting Clinical Outcome with Phenotypic Clusters in COVID-19 Pneumonia: An Analysis of 12,066 Hospitalized Patients from the Spanish Registry SEMI-COVID-19. J. Clin. Med..

[10] Hu B., Huang S., Yin L. (2021). The cytokine storm and COVID-19. J. Med. Virol..

[11] Casas-Rojo J., Antón-Santos J., Millán-Núñez-Cortés J., Lumbreras-Bermejo C., Ramos-Rincón J., Roy-Vallejo E., Artero-Mora A., Arnalich-Fernández F., García-Bruñén J., Vargas-Núñez J. (2020). Clinical characteristics of patients hospitalized with COVID-19 in Spain: Results from the SEMI-COVID-19 Registry. Rev. Clín. Esp..

[12] Horby P., Lim W.S., Emberson J.R., Mafham M., Bell J.L., Linsell L., Staplin N., Brightling C., Ustianowski A., Elmahi E. (2020). Dexamethasone in Hospitalized Patients with Covid-19—Preliminary Report. N. Engl. J. Med..

[13] Rubio-Rivas M., Ronda M., Padulles A., Mitjavila F., Riera-Mestre A., García-Forero C., Iriarte A., Mora J.M., Padulles N., Gonzalez M. (2020). Beneficial effect of corticosteroids in preventing mortality in patients receiving tocilizumab to treat severe COVID-19 illness. Int. J. Infect. Dis..

[14] Stone J.H., Frigault M.J., Serling-Boyd N.J., Fernandes A.D., Harvey L., Foulkes A.S., Horick N.K., Healy B.C., Shah R., Bensaci A.M. (2020). Efficacy of Tocilizumab in Patients Hospitalized with Covid-19. N. Engl. J. Med..

[15] Salvarani C., Dolci G., Massari M., Franco Merlo D., Cavuto S., Savoldi L., Bruzzi P., Boni F., Braglia L., Turrà C. (2021). Effect of Tocilizumab vs Standard Care on Clinical Worsening in Patients Hospitalized With COVID-19 Pneumonia: A Randomized Clinical Trial. JAMA Intern. Med..

[16] Hermine O., Mariette X., Tharaux P.L., Resche-Rigon M., Porcher R., Ravaud P. (2021). CORIMUNO-19 Collaborative Group. Effect of Tocilizumab vs Usual Care in Adults Hospitalized With COVID-19 and Moderate or Severe Pneumonia: A Randomized Clinical Trial. JAMA Intern. Med..

[17] Salama C., Han J., Yau L., Reiss W.G., Kramer B., Neidhart J.D., Criner G.J., Kaplan-Lewis E., Baden R., Pandit L. (2021). Tocilizumab in Patients Hospitalized with Covid-19 Pneumonia. N. Engl. J. Med..

