# Supplementary material for: Risk Categories in COVID-19 Based on Degrees of Inflammation: Data on More Than 17,000 Patients from the Spanish SEMI-COVID-19 Registry
Source: J Clin Med. 2021 May 20;10(10):2214. doi: 10.3390/jcm10102214 (PMC8161115; doi:10.3390/jcm10102214)
Supplement: Supplementary file 1 [file jcm-10-02214-s001.zip › jcm-1161575-supplementary.pdf]

Supplemental files.

Table S1. Outcomes in high-risk patients according to the number of high-risk criteria

	**1 criterion**	**2 criteria**	**3 criteria**	**4 criteria**	**5 criteria**	**p-value**
HFNC, n (%)	319 (6.5)	428 (9.8)	308 (11.2)	197 (15.5)	67 (20.7)	<0.001
NIMV, n (%)	183 (3.7)	279 (6.4)	198 (7.2)	151 (11.9)	46 (14.3)	<0.001
IMV, n (%)	220 (4.4)	352 (8.1)	339 (12.3)	213 (16.8)	71 (22)	<0.001
ICU admission, n (%)	295 (5.9)	436 (9.9)	388 (14)	252 (19.6)	82 (25.2)	<0.001
In-hospital mortality, n (%)	596 (12)	992 (22.6)	907 (32.7)	602 (46.8)	194 (59.5)	<0.001

HFNC: high Flow nasal cannula. NIMV: non-invasive mechanical ventilation. IMV: invasive mechanical ventilation. ICU: intensive care unit.

Table S2. Risk factors of the requirement of HFNC

	**Univariate analysis**		**Multivariate analysis**	
	**OR (95% CI)**	**p-value**	**OR (95% CI)**	**p-value**
Age	1.00 (0.99-1.00)	0.983	-	NS
Gender (female)	0.69 (0.62-0.77)	<0.001	0.82 (0.73-0.92)	0.001
Smoking behaviour			.	NS
Never smoker	1 ref.			
Former smoker	1.42 (1.26-1.60)	<0.001		
Current smoker	1.1.33 (1.06-1.68)	0.016		
Degree of dependency				
None or mild	1 ref.		1 ref.	
Moderate	0.66 (0.53-0.82)	<0.001	0.56 (0.45-0.70)	<0.001
Severe	0.53 (0.41-0.70)	<0.001	0.47 (0.36-0.62)	<0.001
Arterial hypertension	1.14 (1.03-1.27)	0.014	-	NS
Dyslipidemia	1.17 (1.0.5-1.30)	0.005	-	NS
Diabetes mellitus	1.27 (1.12-1.45)	<0.001	1.20 (1.05-1.37)	0.006
Ischemic cardiopathy	1.13 (0.94-1.37)	0.206		
Chronic heart failure	1.04 (0.84-1.28)	0.730		
Chronic liver disease	1.11 (0.85-1.47)	0.438		
Severe chronic renal failure	1.15 (0.93-1.42)	0.208		
Cancer	0.87 (0.72-1.05)	0.140		
COPD	1.62 (1.35-1.95)	<0.001	1.26 (1.04-1.53)	0.017
Asthma	1.09 (0.89-1.33)	0.421		
OSAS	1.77 (1.47-2.13)	<0.001	1.49 (1.23-1.81)	<0.001

Respiratory rate >20 rpm	2.33 (2.09-2.59)	<0.001	2.20 (1.97-2.46)	<0.001
3-risk category				
Low risk	1 ref.		1 ref.	
Intermediate risk	1.43 (0.76-2.66)	0.266	1.31 (0.70-2.45)	0.400
High risk	3.33 (1.82-6.08)	<0.001	2.62 (1.43-4.80)	0.002

NS: Not significant. COPD: chronic obstructive pulmonary disease. HFNC: high flow nasal cannula. OSAS: obstructive sleep apnea syndrome.

Table S3. Risk factors of the requirement of NIMV

	Univariate analysis		Multivariate analysis	
	OR (95% CI)	p-value	OR (95% CI)	p-value
Age	1.00 (0.99-1.00)	0.270	-	NS
Gender (female)	0.65 (0.56-0.74)	<0.001	0.83 (0.72-0.96)	0.011
Smoking behaviour			-	NS
Never smoker	1 ref.			
Former smoker	1.52 (1.32-1.75)	<0.001		
Current smoker	1.35 (1.01-1.79)	0.040		
Degree of dependency				
None or mild	1 ref.		1 ref.	
Moderate	0.64 (0.49-0.83)	0.001	0.49 (0.37-0.64)	<0.001
Severe	0.21 (0.13-0.34)	<0.001	0.18 (0.11-0.29)	<0.001
Arterial hypertension	1.28 (1.12-1.46)	<0.001	-	NS
Dyslipidemia	1.50 (1.31-1.71)	<0.001	1.34 (1.17-1.53)	<0.001
Diabetes mellitus	1.60 (1.38-1.85)	<0.001	-	NS
Ischemic cardiopathy	1.52 (1.24-1.88)	<0.001	-	NS
Chronic heart failure	1.43 (1.14-1.79)	0.002	-	NS
Chronic liver disease	1.22 (0.89-1.68)	0.226		
Severe chronic renal failure	1.18 (0.91-1.52)	0.229		
Cancer	0.93 (0.75-1.16)	0.525		
COPD	2.28 (1.87-2.78)	<0.001	1.52 (1.23-1.88)	<0.001
Asthma	1.08 (0.84-1.38)	0.549		
OSAS	2.82 (2.33-3.42)	<0.001	2.18 (1.78-2.67)	<0.001

Respiratory rate >20 rpm	3.20 (2.80-3.65)	<0.001	2.99 (2.61-3.43)	<0.001
3-risk category				
Low risk	1 ref.		1 ref.	
Intermediate risk	3.59 (1.13-11.39)	0.030	3.13 (0.98-9.98)	0.053
High risk	7.78 (2.49-24.30)	<0.001	5.44 (1.74-17.06)	0.004

NS: Not significant. COPD: chronic obstructive pulmonary disease. NIMV: non-invasive mechanical ventilation. OSAS: obstructive sleep apnea syndrome.

Table S4. Risk factors of the requirement of IMV

	Univariate analysis		Multivariate analysis	
	**OR (95% CI)**	**p-value**	**OR (95% CI)**	**p-value**
Age	0.98 (0.98-0.99)	<0.001	0.99 (0.98-0.99)	<0.001
Gender (female)	0.59 (0.52-0.66)	<0.001	0.71 (0.62-0.80)	<0.001
Smoking behaviour			-	NS
Never smoker	1 ref.			
Former smoker	1.20 (1.06-1.36)	0.006		
Current smoker	1.23 (0.96-1.58)	0.102		
Degree of dependency				
None or mild	1 ref.		1 ref.	
Moderate	0.16 (0.11-0.24)	<0.001	0.19 (0.13-0.29)	<0.001
Severe	0.04 (0.013-0.093)	<0.001	0.04 (0.01-0.10)	<0.001
Arterial hypertension	0.92 (0.82-1.03)	0.157		
Dyslipidemia	1.11 (0.99-1.24)	0.084	1.31 (1.16-1.49)	<0.001
Diabetes mellitus	1.01 (0.88-1.17)	0.876		
Ischemic cardiopathy	0.88 (0.70-1.10)	0.250		
Chronic heart failure	0.35 (0.25-0.50)	<0.001	0.50 (0.35-0.71)	<0.001
Chronic liver disease	0.72 (0.51-1.02)	0.061	-	NS
Severe chronic renal failure	0.43 (0.31-0.61)	<0.001	0.56 (0.40-0.80)	0.001
Cancer	0.62 (0.50-0.77)	<0.001	0.67 (0.53-0.84)	0.001
COPD	0.69 (0.53-0.90)	0.006	0.61 (0.47-0.81)	0.001
Asthma	1.22 (0.99-1.50)	0.064	1.24 (1.01-1.55)	0.049
OSAS	1.34 (1.08-1.67)	0.007	-	NS

Respiratory rate >20 rpm	3.53 (3.14-3.96)	<0.001	3.88 (3.44-4.38)	<0.001
3-risk category				
Low risk	1 ref.		1 ref.	
Intermediate risk	4.78 (1.17-19.52)	0.029	4.50 (1.10-18.44)	0.037
High risk	16.76 (4.17-67.36)	<0.001	14.02 (3.48-56.57)	<0.001

NS: Not significant. COPD: chronic obstructive pulmonary disease. IMV: non-invasive mechanical ventilation. OSAS: obstructive sleep apnea syndrome.

Table S5. Risk factors of ICU admission

	Univariate analysis		Multivariate analysis	
	**OR (95% CI)**	**p-value**	**OR (95% CI)**	**p-value**
Age	0.98 (0.98-0.99)	<0.001	0.98 (0.98-0.99)	<0.001
Gender (female)	0.57 (0.51-0.64)	<0.001	0.71 (0.63-0.80)	<0.001
Smoking behaviour			-	NS
Never smoker	1 ref.			
Former smoker	1.23 (1.10-1.39)	<0.001		
Current smoker	1.37 (1.10-1.70)	0.005		
Degree of dependency				
None or mild	1 ref.		1 ref.	
Moderate	0.17 (0.12-0.25)	<0.001	0.21 (0.15-0.31)	<0.001
Severe	0.04 (0.01-0.08)	<0.001	0.04 (0.02-0.10)	<0.001
Arterial hypertension	0.90 (0.81-0.99)	0.048	1.14 (1.01-1.29)	0.048
Dyslipidemia	1.11 (0.99-1.23)	0.056	1.29 (1.14-1.46)	<0.001
Diabetes mellitus	1.07 (0.94-1.22)	0.285		
Ischemic cardiopathy	0.98 (0.81-1.19)	0.815		
Chronic heart failure	0.40 (0.30-0.53)	<0.001	0.56 (0.41-0.76)	<0.001
Chronic liver disease	0.76 (0.56-1.03)	0.073	-	NS
Severe chronic renal failure	0.50 (0.37-0.66)	<0.001	0.64 (0.48-0.87)	0.004
Cancer	0.62 (0.51-0.76)	<0.001	0.67 (0.54-0.83)	<0.001
COPD	0.75 (0.59-0.94)	0.013	0.63 (0.49-0.82)	<0.001
Asthma	1.24 (1.03-1.50)	0.023	1.27 (1.04-1.55)	0.017
OSAS	1.36 (1.12-1.65)	0.002	-	NS

Respiratory rate >20 rpm	3.24 (2.92-3.61)	<0.001	3.60 (3.22-4.02)	<0.001
3-risk category				
Low risk	1 ref.		1 ref.	
Intermediate risk	4.36 (1.38-13.81)	0.012	4.27 (1.34-13.55)	0.014
High risk	13.75 (4.41-42.91)	<0.001	12.29 (3.92-38.49)	<0.001

NS: Not significant. COPD: chronic obstructive pulmonary disease. ICU: intensive care unit. OSAS: obstructive sleep apnea syndrome.

Table S6. Matrix of correlations between prognostic factors.

	Age	Sex	Smoking behavior	Degree of dependancy	Arterial hypertension	Dyslipemia	Diabetes mellitus	Ischaemic cardiopathy	Chronic heart failure	Chronic liver disease	Severe chronic renal failure	Cancer	COPD	Asthma	OSAS	Tachypnea
Age	1	0.065	0.017	0.385	0.451	0.276	0.180	0.160	0.225	0.014	0.153	0.114	0.150	-0.051	0.024	0.134
Sex	0.065	1	-0.273	0.110	<0.001	<0.001	-0.042	-0.094	0.011	-0.048	-0.021	-0.046	-0.136	0.097	-0.084	-0.034
Smoking behavior	0.017	-0.273	1	-0.060	0.066	0.085	0.062	0.106	0.053	0.086	0.045	0.086	0.299	-0.016	0.112	0.051
Degree of dependancy	0.385	0.110	-0.060	1	0.150	0.035	0.092	0.043	0.178	0.012	0.107	0.032	0.024	-0.046	-0.021	0.107
Arterial hypertension	0.451	<0.001	0.066	0.150	1	0.337	0.252	0.171	0.178	0.038	0.175	0.053	0.098	-0.024	0.102	0.083
Dyslipemia	0.276	<0.001	0.085	0.035	0.337	1	0.248	0.203	0.099	0.029	0.108	0.032	0.079	-0.020	0.077	0.053
Diabetes mellitus	0.180	-0.042	0.062	0.092	0.252	0.248	1	0.147	0.109	0.072	0.148	0.039	0.061	-0.032	0.089	0.056
Ischaemic cardiopathy	0.160	-0.094	0.106	0.043	0.171	0.203	0.147	1	0.198	0.026	0.101	0.039	0.096	-0.022	0.050	0.045
Chronic heart failure	0.225	0.011	0.053	0.178	0.178	0.099	0.109	0.198	1	0.061	0.170	0.036	0.115	-0.002	0.066	0.082
Chronic liver disease	0.014	-0.048	0.086	0.012	0.038	0.029	0.072	0.026	0.061	1	0.065	0.043	0.064	-0.006	0.029	0.008
Severe chronic renal failure	0.153	-0.021	0.045	0.107	0.175	0.108	0.148	0.101	0.170	0.065	1	0.056	0.065	-0.020	0.018	0.039
Cancer	0.114	-0.046	0.086	0.032	0.053	0.032	0.039	0.039	0.036	0.043	0.056	1	0.060	-0.031	0.012	0.009
COPD	0.150	-0.136	0.299	0.024	0.098	0.079	0.061	0.096	0.115	0.064	0.065	0.060	1	-0.012	0.125	0.095
Asthma	-0.051	0.097	-0.016	-0.046	-0.024	-0.020	-0.032	-0.022	-0.002	-0.006	-0.020	-0.031	-0.012	1	0.041	0.004
OSAS	0.024	-0.084	0.112	-0.021	0.102	0.077	0.089	0.050	0.066	0.029	0.018	0.012	0.125	0.041	1	0.048
Tachypnea	0.134	-0.034	0.051	0.107	0.083	0.053	0.056	0.045	0.082	0.008	0.039	0.009	0.095	0.004	0.048	1
